# Preoperative cardiac troponin level is associated with all-cause mortality of liver transplantation recipients

**DOI:** 10.1371/journal.pone.0177838

**Published:** 2017-05-23

**Authors:** Jungchan Park, Seung Hwa Lee, Sangbin Han, Hyun Sook Jee, Suk-Koo Lee, Gyu-Seong Choi, Gaab Soo Kim

**Affiliations:** 1 Department of Anesthesiology and Pain Medicine, Samsung Medical Center, Sungkyunkwan University School of Medicine, Seoul, Korea; 2 Division of Cardiology, Department of Medicine, Heart Vascular Stroke Institute, Samsung Medical Center, Sungkyunkwan University School of Medicine, Seoul, Korea; 3 Department of Surgery, Samsung Medical Center, Sungkyunkwan University School of Medicine, Seoul, Korea; University of Colorado, UNITED STATES

## Abstract

This study was aimed to evaluate the association between preoperative high-sensitivity cardiac troponin I (hs-cTnI) level and mortality in patients undergoing liver transplantation (LT). From January 2011 to May 2016, preoperative hs-cTnI level was measured in consecutive 487 patients scheduled for LT. Patients with elevated preoperative hs-cTnI were compared with those who had normal level. The primary outcome was all-cause death in follow-up period of 30 days to 1 year after operation. Of the 487 patients, 58 (11.9%) had elevated preoperative hs-cTnI and 429 (88.1%) had normal preoperative hs-cTnI. In multivariate analysis, the rate of 1-year mortality and 30-day mortality were higher in elevated preoperative hs-cTnI group (hazard ratio [HR], 3.69; confidence interval [CI] 95%, 1.83–7.42; p < 0.001, HR, 6.61; CI, 1.91–22.82; p = 0.003, respectively). After adjustment with inverse probability weighting (IPW), the incidence of 1-year mortality and 30-day mortality were higher in elevated group (HR, 4.66; CI, 3.56–6.1; p < 0.001, HR, 10.31; CI, 6.39–16.66; p < 0.001, respectively). In conclusion, this study showed that in patients who underwent LT, elevation of preoperative hs-cTnI level was associated with 1-year mortality and 30-day mortality.

## Introduction

Cardiac troponin is a contractile protein located within myocyte and known as a diagnostic test of stress on the heart which is closely related to perioperative mortality [1]. Moreover, the new-generation cardiac troponin test with high sensitivity not only lowered the number of potentially missed coronary artery disease (CAD) patients but also has prognostic value in diseases that do not originate in the coronary arteries including congestive heart failure, pulmonary embolism and chronic kidney disease [2–4]. This enabled cardiac troponin to provide prognostic information of all-cause death during perioperative period among patients undergoing non-cardiac surgery [5].

As liver transplantation (LT) has become a successful treatment option for patients with end-stage liver disease, effective screening test to distinguish high risk candidates is required concerning disparity between LT necessity and organ availability [6]. Up to this date, limited studies have referred predictive value of preoperative cardiac troponin in patients undergoing LT and those studies have focused on cardiac events among relatively small number of patients without clear reference limit according to 99^th^ percentile rule which was proposed as a myocardial injury regardless of ischemic etiology [7–9]. We hypothesized that preoperative high-sensitivity cardiac troponin I (hs-cTnI) is associated with all-cause mortality within 1-year follow-up in patients undergoing LT.

## Methods

### Study population and data collection

The present study was a single-center study. The study population was selected from the LT database of our hospital. Our hospital is an experienced large-volume center located in Seoul, South Korea. For nearly 20 years, over 1800 LT were performed in our hospital. From January 2011 to May 2016, consecutive 646 recipients were enrolled into our registry. The inclusion criteria were patients with preoperative hs-cTnI test before LT. The exclusion criteria were: 1) patients age under 18 years old; 2) patients with known CAD or heart failure; 3) patients with follow-up loss. Clinical, laboratory and outcome data up to 1-year follow-up were collected by a trained study coordinator using a standardized case report form and protocol. The study protocol was approved by the Institutional Review Board of Samsung medical center (IRB file number: 2016-07-161-001). All participants were accessed anonymously for analysis and consents from participants were waived by Institutional Review Board.

### Anesthetic management

Standardized anesthesia was performed in accordance with our institutional LT protocol. The standard monitoring devices (peripheral capillary oxygen saturation, 5-lead electrocardiography, non-invasive arterial blood pressure) were applied and anesthesia was induced with thiopental sodium (5 mg/kg) and maintained with isoflurane titrated to a bispectral index of 40–60. Remifentanil was also infused up to 0.20 mcg/kg/min according to hemodynamic responses. Mechanical ventilation was set at a tidal volume of 8–10 ml/kg using a mixture of medical air and oxygen at a fresh gas flow rate of 2 L/min with respiratory rate adjusted to maintain normocapnea. The radial artery, femoral artery, femoral vein, and internal jugular vein were cannulated for direct hemodynamic monitoring. Infusions of fluids and vasoactive drugs, such as dopamine and norepinephrine, were aimed to maintain mean arterial pressure ≥ 70 mmHg. A warm blanket and a fluid warmer were used to maintain normothermia with room temperature thermostatically set at 24°C. Packed red blood cells were transfused when blood hemoglobin concentration was < 8.0 mg/dL.

### Preoperative hs-cTnI level

All patients scheduled for LT underwent standardized preoperative evaluation which includes hs-cTnI test according to protocol of transplant unit in our institution. Preoperative hs-cTnI level was assessed as a single time point analysis within preoperative evaluation which was done one day prior to the operation in most of cases except in highly emergent situations related to acute liver failure. With highly sensitive immunoassay, hs-cTnI was measured by means of an automated analyzer (Advia Centaur XP, Siemens Healthcare Diagnostics, Erlangen, Germany). Lower limit of detection was 0.006 ng/mL and normal range was 0.04 ng/mL according to 99th percentile reference [10].

### Definition and outcomes

Diabetes mellitus was defined as having a history of type 1 or type 2 diabetes mellitus or hemoglobin A1c > 6.5% or fasting blood glucose > 126 mg/dL on 2 separate occasions. Hypertension was defined as either self-reported antihypertensive medications or systolic blood pressure >140 mm Hg. Ascites was detected immediately after surgical insicion. Preoperative hemoglobin was measured on routine preoperative evaluation along with hs-cTnI. Preoperative transfusion was a history of packed red blood cell transfusion within two days before LT. The primary endpoint was all-cause death within the follow-up period which was from 30 days up to 1 year from LT. The secondary endpoint was 30-day mortality.

### Statistical analysis

Continuous variables were compared by using the t-test or the Wilcoxon rank-sum test when applicable and presented as mean ± standard deviation (SD). Categorical data of each group were evaluated using Chi-square or Fisher’s exact test. Survival curves were constructed using Kaplan-Meier estimates and compared with the log-rank test. Covariates with a univariable effect on mortality with a p value < 0.1 or clinically relevant were retained in the multivariable model. Adjustment was made for the following baseline variables: female gender, age, deceased type of donor, acute hepatic failure, varix, encephalopathy, ascites, diabetes mellitus, smoking, chronic kidney disease, dialysis, preoperative hemoglobin, preoperative transfusion, model for end stage liver disease with sodium (MELD-Na) score. Multiple Cox regression analysis was done to identify independent predictor. Variables included in analysis were deceased type donor, diabetes mellitus, encephalopathy, preoperative transfusion, intraoperative dopamine use and intraoperative norepinephrine use. Hazard ratios (HR) are reported with 95% confidence intervals (CI). To retain a large sample size and maximize the study power while maintaining a balance in covariates between two groups, we conducted rigorous adjustment for differences in baseline characteristics of patients using the weighted Cox proportional-hazards regression models with the inverse probability weighting (IPW) [11]. According to this technique, weights for patients with elevated hs-cTnI were the inverse of the propensity score and weights for patients with normal hs-cTnI were the inverse of 1 –the propensity score. The propensity scores were estimated without regard to outcomes, using multiple logistic-regression analysis. The reduction in the risk of outcome was compared using the stratified Cox regression model. Statistical analyses were performed with SAS 9.4 (SAS Institute Inc., Cary, NC, USA). All tests were 2-tailed and p < 0.05 was considered statistically significant.

## Result

A total of 646 LT were performed from January 2011 to May 2016. 90 recipients were excluded for being younger than 18 years old. 63 recipients with missing value of preoperative hs-cTnI were also excluded from study along with 3 recipients due to prior CAD or heart failure and 3 recipients due to follow-up loss. 487 recipients were left for analysis. The mean duration from hs-cTnI evaluation to LT was 23.9 hours in normal group and 17.6 hours in elevated group (p = 0.6).

### Baseline characteristics

A total of 487 patients were divided into two groups: 58 (11.9%) in the elevated hs-cTnI group and 429 (88.1%) in the normal hs-cTnI group. The baseline characteristics are summarized in Table 1. The recipient`s mean age was 55 years (range from 19 to 80). Patients with preoperative hs-cTnI elevation showed higher frequency of female gender and of deceased type of donor (44.8% vs 22.4% p < 0.001, 55.2% vs 21.2% p < 0.001, respectively) compared to the normal hs-cTnI group. Acute hepatic failure (15.5% vs 2.6% p < 0.001) and encephalopathy (43.1% vs 9.6% p < 0.001) were also more common in elevated group. Additionally, the elevated hs-cTnI group showed a higher MELD-Na score (29.3 vs 17.1 p < 0.001) and lower preoperative hemoglobin level (9.5 vs 10.8 p < 0.001) compared to the normal hs-cTnI group.

**Table 1:** Baseline characteristics.

	Elevated troponin % (N = 58)	Normal troponin % (N = 429)	p value	IPW p value
Female	44.8 (26)	22.4 (96)	< 0.001	0.1
Age	55.7 (±10.7)	54.9 (±8.79)	0.61	0.52
Deceased donor	55.2 (32)	21.2 (91)	< 0.001	0.04
Acute hepatic failure	15.5 (9)	2.6 (11)	< 0.001	0.53
Varix	37.9 (22)	46.9 (201)	0.2	0.49
Encephalopathy	43.1 (25)	9.6 (41)	< 0.001	< 0.001
Ascites	63.8 (37)	43.1 (185)	0.003	0.86
Diabetes	24.1 (14)	24.7 (106)	0.92	0.76
Hypertension	17.2 (10)	14.0 (60)	0.51	0.86
Smoking	32.8 (19)	38.0 (163)	0.44	0.33
Chronic kidney disease	3.4 (2)	0.5 (2)	0.07	0.32
Dialysis	1.7 (1)	0.5 (2)	0.32	0.65
Chronic alcoholics	43.1 (25)	44.1 (189)	0.89	0.68
Stroke	1.7 (1)	0.5 (2)	0.32	0.15
Preop hemoglobin	9.5 (±1.7)	10.8 (±2.3)	< 0.001	0.22
Preop transfusion	25.9 (15)	5.6 (24)	< 0.001	0.01
Introp Dopamine>5mcg/kg/min	82.8 (48)	79.7 (342)	0.59	0.65
Introp Noreipnephrine>0.05mcg/kg/min	74.1 (43)	68.8 (295)	0.4	0.65
MELD-Na score	29.3 (±11.5)	17.1 (±11.4)	< 0.001	< 0.001
Total bilirubin	19.64 (±16)	7.3 (±11.63)	< 0.001	
INR	3 (±1.33)	1.92 (±2.58)	0.002	
Creatinine	1.36 (±0.9)	1.1 (±0.74)	0.017	
Sodium	137 (±9)	138 (±6)	0.03	

Values are mean (±standard deviation) or % (n); MELD-Na, model for end stage liver disease with Sodium; INR, international normalized ratio

### Clinical outcomes

**Fig 1:**
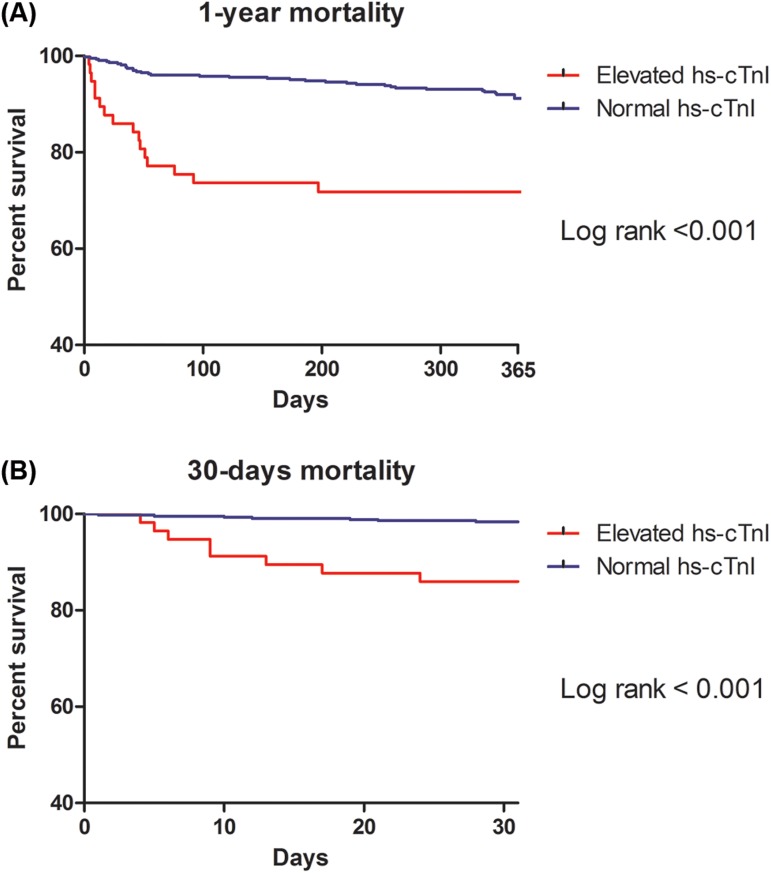
The Kaplan-Meier Curves for the hs-cTnI elevated and normal hs-cTnI group. Curves for (A) all-cause death in 1-year, and (B) all-cause death in 30 days.

**Fig 2:**
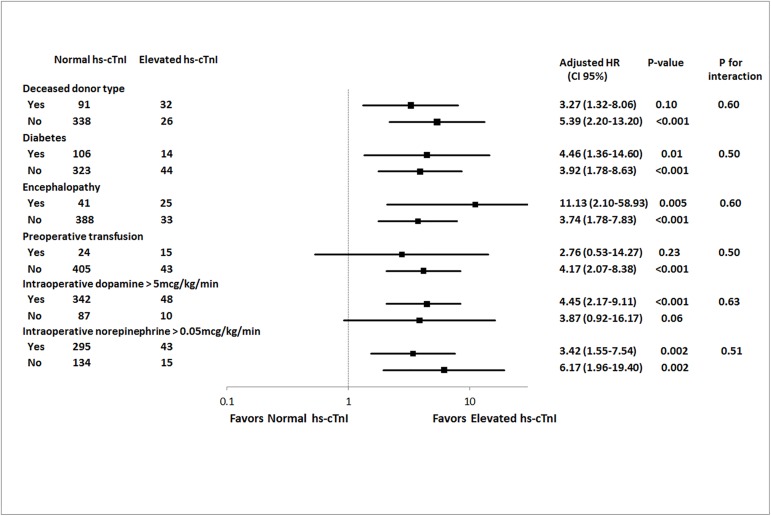
Subgroup analysis of deceased donor type, diabetes mellitus, encephalopathy, preoperative transfusion, intraoperative dopamine use and intraoperative norepinephrine use for all-cause death in 1-year.

Survival curves estimating 1-year mortality and 30-day mortality is shown in Fig 1. The median postoperative follow-up period was 365 days (365days-365days). Clinical outcomes are shown in Table 2. In univariate analysis, age, deceased type of donor, varix, diabetes mellitus, smoking, preoperative transfusion, MELD-Na score and preoperative hs-cTnI were associated with mortality within follow-up period up to 1 year. 30-day mortality was associated with deceased type of donor, chronic kidney disease, dialysis, preoperative hemoglobin, MELD-Na score and preoperative hs-cTnI. In multivariate analysis within follow-up period up to 1 year and 30 days, patients with preoperative hs-cTnI elevation had a higher risk of all-cause death (HR, 3.69; CI, 1.83–7.42, p < 0.001, HR, 6.61; CI, 1.91–22.82, p = 0.003, respectively) than those in the normal group. In addition, the mortality up to 1 year and 30 days were also higher in the hs-cTnI elevated group based on adjustment with IPW (HR, 4.66; CI, 3.56–6.1, p < 0.001, HR, 10.31; CI, 6.39–16.66, p < 0.001, respectively). In subgroup analysis, no variables showed a interaction and the results are shown in Fig 2.

**Table 2:** Preoperative variables associated with clinical outcome.

	Univariate Analysis		Multivariate Analysis		IPW analysis	
	HR (CI 95%)	p value	HR (CI 95%)	p value	HR (CI 95%)	p value
Death in a year (n = 56)						
^*^Female	1.01 (0.55–1.86)	0.97	1.07 (0.51–2.22)	0.86		
^*^Age	1.04 (1.01–1.07)	0.02	1.04 (1.01–1.07)	0.01		
^*^Deceased donor	2.37 (1.39–4.02)	0.001	1.3 (0.66–2.55)	0.45		
^*^Acute hepatic failure	0.9 (0.22–3.69)	0.88	0.44 (0.09–2.17)	0.32		
^*^Varix	0.57 (0.33–1.0)	0.05	0.57 (0.31–1.05)	0.07		
^*^Encephalopathy	1.45 (0.73–2.87)	0.29	0.63 (0.29–1.39)	0.26		
^*^Ascites	1.31 (0.78–2.21)	0.31	0.9 (0.48–1.68)	0.74		
^*^Diabetes	1.87 (1.09–3.21)	0.02	1.41 (0.78–2.53)	0.26		
Hypertension	1.36 (0.69–2.7)	0.38				
^*^Smoking	1.7 (1.0–2.87)	0.05	1.83 (0.96–3.47)	0.07		
^*^Chronic kidney disease	2.47 (0.34–17.82)	0.37	0.29 (0.03–3.07)	0.3		
^*^Dialysis	4.01 (0.55–28.97)	0.17	8.07 (0.76–85.6)	0.08		
Chronic alcoholics	1.03 (0.61–1.74)	0.93				
Stroke	0.05 (0–1751)	0.69				
^*^Preop hemoglobin	0.94 (0.83–1.05)	0.27	0.99 (0.86–1.15)	0.91		
^*^Preop transfusion	2.84 (1.43–5.63)	0.03	1.22 (0.53–2.77)	0.64		
^*^MELD-Na score	1.03 (1.01–1.05)	0.001	1.02 (0.99–1.05)	0.24		
Introp Dopamine>5mcg/Kg/min	0.77 (0.41–1.43)	0.4				
Introp Noreipnephrine>0.05mcg/Kg/min	0.95 (0.54–1.66)	0.85				
Elevated Troponin	4.65 (2.67–8.1)	< 0.001	3.69 (1.83–7.42)	< 0.001	4.66 (3.56–6.1)	<0.001
Death in 30 days (n = 16)						
^*^Female	2.35 (0.0.88–6.31)	0.09	1.98 (0.48–8.18)	0.35		
^*^Age	1.03 (0.97–1.09)	0.28	1.03 (0.98–1.09)	0.22		
^*^Deceased donor	5.1 (1.85–14.04)	0.002	1.92 (0.55–6.74)	0.31		
^*^Acute hepatic failure	1.6 (0.21–12.11)	0.65	0.6 (0.05–6.74)	0.31		
^*^Varix	0.54 (0.19–1.54)	0.25	0.83 (0.23–3.01)	0.78		
^*^Encephalopathy	1.49 (0.43–5.24)	0.53	0.41 (0.09–1.8)	0.24		
^*^Ascites	1.2 (0.45–3.19)	0.72	0.37 (0.11–1.19)	0.09		
^*^Diabetes	1.4 (0.49–4.04)	0.53	0.92 (0.24–3.51)	0.9		
Hypertension	1.99 (0.64–6.17)	0.23				
^*^Smoking	1.32 (0.49–3.53)	0.59	3.15 (0.72–13.72)	0.13		
^*^Chronic kidney disease	8.33 (1.1–63.09)	0.04	0.26 (0.02–4.56)	0.36		
^*^Dialysis	11.32 (1.49–85.82)	0.02	53.29 (2.46–1153.7)	0.01		
Chronic alcoholics	0.77 (0.28–2.11)	0.61				
Stroke	0.05 (0–5941)	0.83				
^*^Preop hemoglobin	0.78 (0.61–1.0)	0.05	0.93 (0.67–1.28)	0.64		
^*^Preop transfusion	2.69 (0.77–9.45)	0.12	0.56 (0.12–2.7)	0.47		
^*^MELD-Na score	1.06 (1.03–1.1)	< 0.001	1.06 (1.01–1.11)	0.03		
Introp Dopamine>5mcg/Kg/min	1.77 (0.4–7.79)	0.45				
Introp Noreipnephrine>0.05mcg/Kg/min	0.73 (0.27–2.01)	0.54				
Elevated Troponin	10.30 (3.83–27.66)	< 0.001	6.61 (1.91–22.82)	0.003	10.31 (6.39–16.66)	< 0.001

Values are mean (±standard deviation); MELD-Na, model for end stage liver disease with sodium; INR, international normalized ratio

^*^Covariates include Female, age, deceased donor, acute hepatic failure, varix, encephalopathy, ascites, diabetes, smoking, chronic kidney disease, dialysis, preoperative hemoglobin, preoperative transfusion and MELD-Na score

## Discussion

The result of present study demonstrates that preoperative elevation of hs-cTnI is associated with postoperative mortality within 1-year follow-up in patients undergoing LT. This finding indicates that preoperative hs-cTnI may be helpful in predicting high risk LT candidates. Considering a recent study showing that longer time from elevated hs-cTnI to surgery reduces postoperative mortality, delaying LT may be considered after further studies [12].

Three subunits (T, I, and C) of cardiac troponin are the products of different genes. Both troponin T and I are ideally suited for the detection of myocardial damage as they are expressed as cardio-specific isoforms [9]. Among T and I isoform of cardiac troponin, the T isoform was most commonly used for CAD diagnosis. But the I isoform is just as sensitive but less affected by renal dysfunction than T isoform [4]. The I isoform was selected in this study.

Traditionally cardiac troponin has been accepted as a well-established biomarker with both diagnostic and prognostic value in CAD. In addition, cardiac troponin has subsequently been analyzed in a wide variety of settings other than cardiac conditions as an indicator of metabolic stress and associated with an adverse outcome in most of studies with non-cardiac conditions [5]. Recently, evolution of high-sensitivity assays widened its predictive value in clinical outcomes among patients with stable CAD and a reference limit according to 99^th^ precentile rule has been promoted to detect myocardial injury regrdless of ischemic cause [9,13]. As an isolated hs-cTnI elevation above reference limit is present in patients under metabolic stress, it may be suggestive but clearly not indicative of CAD and it may present in patients under metabolic stress. Many studies have been conducted on elevation of hs-cTnI in patients with various non-coronary diseases and even in nonhospitalized population without overt illness [14–16]. Therefore, all-cause mortality within 1-year follow-up was analyzed as a primary end point in this study. Elevation of hs-cTnI after surgery has been reported to be associated with postoperative myocardial infarction in non-cardiac surgery [17–19]. However, most of previous studies have focused on elevation of postoperative hs-cTnI level and routine follow-up in postoperative period is still on debate [17].

Elevation of cardiac troponin was strongly associated with risk of cardiovascular events, mortality and even with graft loss in LT [6–8]. An association of preoperative hs-cTnI with mortality and cardiovascular events among 230 patients undergoing LT was evaluated and showed significant result in 1-year and 5-year follow-up [6]. Other study showed that history of cardiovascular disease and hs-cTnI predicts patient and graft survival following LT [7]. However, neither of these studies were performed with a 99th percentile cut-off value. Recent study among 78 LT recipients showed significant difference of mortality between normal, intermediate and elevated cardiac troponin T groups [8]. This study was also done with relatively smaller number of subjects without excluding patients with prior CAD. Up to this date, limited data referring an elevation of clearly defined 99^th^ percentile value of preoperative hs-cTnI and postoperative overall mortality in shorter than 1-year follow-up among LT recipients exist. To evaluate overall mortality while minimizing influence of underlying cardiac conditions, patients with previous CAD or heart failure were excluded in our study. By this means, hs-cTnI is assumed to be associated with adverse outcomes related to not only CAD, but also general status. Pediatric patients in which normal level of hs-cTnI is still on debate were excluded to use 99th percentile cut-off value of new-generation hs-cTnI [20].

Considering shortage of organ availability, screening high-risk candidates for LT is increasingly more important. The MELD score has traditionally been accepted as the standard method for organ allocation for LT and preoperative sodium level has also been suggested along with MELD score (MELD-Na) to improve accuracy of survival prediction [21,22]. Perioperative risk due to severe hemodynamic change during LT has led to the development of a variety of scoring systems for cardiac risk stratification in the last decade. There still remain wide variations in the center specific protocols [23]. Use of hs-cTnI assay as a test for risk stratification requires only a single and relatively inexpensive blood test which is widely available and routinely performed in many institutes. A clearly defined ‘cut-off point’, such as 0.04 ng/mL according to 99th percentile reference limit would be simple to use in a clinical setting without the difficulties in applying complex scoring systems to individual patients nor potential for subjective interpretation of clinical parameters. By integrating with preoperative evaluation, more caution could be used even before the transplantation procedure in those with expected adverse outcome related to cardiac and non-cardiac conditions.

This study has advantages over previous studies. To focus on hs-cTnI elevation caused by other than ischemic etiology, patients with history of heart disease was excluded and overall mortality was analyzed as a primary endpoint. By this means, predictive value of hs-cTnI in LT may be widened beyond cardiac morbidity. IPW analysis was used to minimize biased estimates. The use of IPW has rapidly increased in recent years [11].

The limitations of this study include the nature of non-randomized and observational study which could have significantly affected the results due to confounding factors. Although an IPW analysis was performed to adjust for these potential confounding factors, unmeasured variables were not able to be corrected. Considering that rise or fall in serial hs-cTnI levels is a critical component of diagnosis, an absolute level of hs-cTnI in this study might be insufficient. Another limitation is that this study was a single center study in Asia. Most of recipients were Asians except two recipients from Middle East. Ethnic difference of normal hs-cTnI level which might have affected the results was not included in this analysis due to small number [24]. Despite of these, by evaluating influence of preoperative hs-cTnI, this study may be helpful in risk assessment and prediction of prognosis in LT recipients. A further study is required to determine whether delaying LT in patients with elevated hs-cTnI is necessary.

## Conclusion

Preoperative hs-cTnI is strongly associated with postoperative mortality after LT within 1-year follow-up. Further study referring perioperative hs-cTnI level and its kinetics is required.

## Supporting information

S1 Table Minimal dataset of all participants. (XLSX)

## Abbreviations

LT: liver transplantation
Hs-cTnI: high-sensitivity cardiac troponin
CAD: coronary artery diseasec
MELD-Na: model for end stage liver disease with sodium
